# Virtual Stiffness: A Novel Biomechanical Approach to Estimate Limb Stiffness of a Multi-Muscle and Multi-Joint System

**DOI:** 10.3390/s23020673

**Published:** 2023-01-06

**Authors:** Daniele Borzelli, Stefano Pastorelli, Andrea d’Avella, Laura Gastaldi

**Affiliations:** 1 Department of Biomedical and Dental Sciences and Morphofunctional Imaging, University of Messina, 98122 Messina, Italy; 2 Laboratory of Neuromotor Physiology, IRCCS Santa Lucia Foundation, 00179 Rome, Italy; 3 Department of Mechanical and Aerospace Engineering, Politecnico di Torino, 10129 Turin, Italy

**Keywords:** myoelectric control, impedance estimation, real-time control, null-space control, EMG-to-force mapping, musculoskeletal model, muscle redundancy, exoskeleton

## Abstract

In recent years, different groups have developed algorithms to control the stiffness of a robotic device through the electromyographic activity collected from a human operator. However, the approaches proposed so far require an initial calibration, have a complex subject-specific muscle model, or consider the activity of only a few pairs of antagonist muscles. This study described and tested an approach based on a biomechanical model to estimate the limb stiffness of a multi-joint, multi-muscle system from muscle activations. The “virtual stiffness” method approximates the generated stiffness as the stiffness due to the component of the muscle-activation vector that does not generate any endpoint force. Such a component is calculated by projecting the vector of muscle activations, estimated from the electromyographic signals, onto the null space of the linear mapping of muscle activations onto the endpoint force. The proposed method was tested by using an upper-limb model made of two joints and six Hill-type muscles and data collected during an isometric force-generation task performed with the upper limb. The null-space projection of the muscle-activation vector approximated the major axis of the stiffness ellipse or ellipsoid. The model provides a good approximation of the voluntary stiffening performed by participants that could be directly implemented in wearable myoelectric controlled devices that estimate, in real-time, the endpoint forces, or endpoint movement, from the mapping between muscle activation and force, without any additional calibrations.

## 1. Introduction

In the 1980s, the importance of impedance modulation for a robotic device that interacts with a perturbative environment was stated by Hogan [1]. Since then, a variety of variable impedance actuators have been developed [2]. However, even with variable impedance actuators that can exert high stiffness, no robotic devices could achieve the superior motor capabilities of a human. A human can perform an effective and flexible modulation of both motion and impedance in a smooth and efficient manner according to the environment and the required task. Therefore, it was proposed to control the impedance of a robotic device through the real-time estimation of the impedance exerted by a human operator [3,4], thus combining the high stiffening capabilities of the robot with the high control capabilities of the human operator.

However, this procedure raises the issue of how to estimate, in real-time, the stiffness exerted by the operator. Perturbation-based techniques [5] directly measuring the restoring force after small imposed displacements are currently the most accurate and reliable methods for impedance estimation. Nevertheless, their application may be problematic when the estimation of the human arm impedance is required in real-time, during the execution of a task, because the applied external perturbation may be deleterious for the task. While several studies investigated how to detect the rotational stiffness of lower-limb joints during locomotion, exploiting the cyclicity of gait [6] or implementing musculoskeletal models driven by the muscle activity [7], less attention has been given to the real-time estimation of upper-limb stiffness modulation during non-cyclic movements. Since joint-stiffness changes are due to muscles’ co-activation [8], whose modulation does not lead to kinematic variations or to joint moments, the stiffness of a joint or of a whole limb is commonly estimated from the electromyographic (EMG) signals. EMG magnitude was observed to be strongly correlated both with the endpoint arm stiffness and the endpoint force [9]. Simple methods were proposed for estimating the stiffness of a single joint, e.g., the knee [10] or the elbow [11], based on the activation of a limited set of pairs of antagonist muscles. However, the estimation of the endpoint stiffness, generated by a highly redundant musculoskeletal system through the activity of a limited number of muscles may be inaccurate. Moreover, the agonist/antagonist definitions are oversimplified, especially in the presence of multi-articular muscles [12]. 

Approaches for the estimation of the stiffness of a limb exploiting musculoskeletal models were recently implemented in real-time applications [13,14]. However, musculoskeletal models may not be optimal for all applications, because they require subject-specific parameters. While the scaling of a standard model based only on the subject’s height, weight, and segments’ length was proposed [15], the accuracy and the precision of the model predictions may not be adequate for all applications [16], and customized solutions may be required [17]. Moreover, the implementation of these models is computationally expensive and provides limited support for contact-rich interactions [18].

An estimation of the mapping between EMG and joint torque was proposed by Ajoudani and collaborators [3] for tele-impedance applications, based on an initial calibration. However, this calibration may not be consistent along different experimental sessions, and longitudinal experiments may require a different calibration for each session.

An elegant approach that detected co-contraction during locomotion after the calculation of the Continuous Wavelet Transform [19] was recently proposed. This approach did not only determine the time bins, but also the frequency bands at which two muscles are simultaneously activated. However, this approach did not provide an estimation of the stiffness level, and it was designed for pairs of muscles. 

In sum, methods for the estimation of the endpoint stiffness when several muscles act on multiple joints are still missing, and a solution with a low computational cost that provides a consistent estimation of the stiffness generated by several muscles acting on multiple joints, without reducing the musculoskeletal redundancy, is still missing. 

In this study, we described and tested a simple biomechanical approach, called “virtual impedance” [20,21], to estimate the stiffness generated by several muscles acting on multiple joints. In contrast to existing approaches, we investigated the co-contraction of several muscles at the same time, without any foreknown knowledge of the group of muscles that are expected to co-contract. The proposed method approximates the limb stiffness with a term proportional to the norm of the component of the muscle activation vector that does not generate any endpoint force, i.e., the null-space projection of the muscle activation vector [22]. The method was tested by using an arm model composed of six muscles acting on two joints and experimental data collected during an upper-limb isometric force exertion task. Since it was already demonstrated that healthy participants are able to modulate the norm of this null-space projection during isometric force generation [20] and to selectively modulate only some components of this null-space projection [23], here we propose to use null-space projections of EMG signals to control the stiffness of tele-operated robots. However, the simplicity of the proposed method makes it a valid candidate for the stiffness control of wearable robotic devices, such as exoskeletons and prostheses, and for motor augmentation. The advantages of this method are its low computational cost; ease of implementation, as it does not require any additional calibration once the mapping between EMG and force is determined; and its consistency across multiple experimental sessions.

## 2. Materials and Methods

In this study, we tested whether the norm of the null-space component of the muscle activation may approximate the stiffness exerted by a musculoskeletal system both on simulated data that were generated by a musculoskeletal model made of two joints and six muscles and on experimental data that were collected from twelve upper-limb muscles. 

### 2.1. Upper-Limb Musculoskeletal Simulation

In this section, we describe the musculoskeletal simulation that was implemented to validate the proposed approach.

#### 2.1.1. Upper-Limb Model

The model is composed of two joints and six muscles acting on them. The elbow is at the same height as the shoulder, and the arm is assumed to lie on a rigid horizontal surface; therefore, no muscle action for gravity compensation is required. The arm is assumed to generate forces only along the horizontal plane. The parameters were selected to approximate the elbow and shoulder of the upper limb, but the same model with different parameters could approximate other pairs of joints, such as the ankle and the knee, or the knee and the hip.

**Figure 1:**
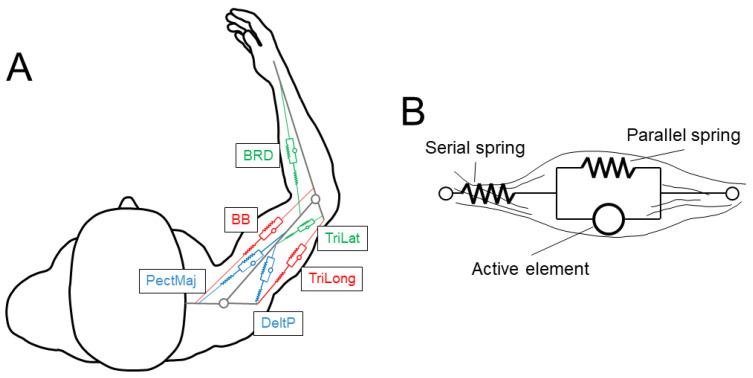
(**A**). The elbow–shoulder musculoskeletal model comprising 2 joints and 6 muscles (shoulder monoarticular in blue, elbow monoarticular in green, and biarticular in red). (**B**). The Hill muscle model, which approximated each muscle, is composed of three elements: an active element, a spring in parallel with the active element, and a spring in series with the active element.

**Figure 2:**
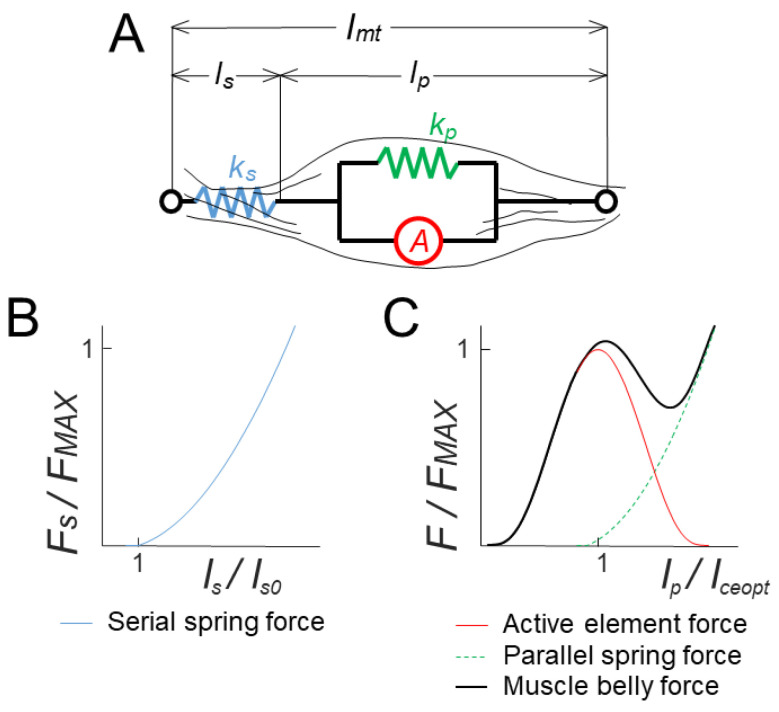
The Hill musculotendon model. (**A**). The musculotendon system is modeled with an active element A and a parallel spring (*k_p_*), which modulates the action of the muscle belly fibers, and a serial spring (*k_s_*), which modulates the tendons action. (**B**). The force exerted by the serial spring, depending on the relative length of the tendons (*l_s_/l_s*0*_*). (**C**). The force exerted by the muscle belly (black) as the sum of the action exerted by the parallel spring (green) and by the active element (red). The magnitude of the exerted force depends on the relative length of the belly (*l_p_/l_ceopt_*).

The 6 muscles (see Figure 1A) were selected as in [9]. There were two mono-articular muscles acting on the elbow joint (brachioradialis, BRD, a flexor; and the lateral head of triceps brachii, TriLat, an extensor), two mono-articular muscles acting on the shoulder joint (pectoralis major sternal, PecMaj, a flexor; and the posterior deltoid, DeltP, an extensor), and two bi-articular muscles acting both on the shoulder and the elbow joints (biceps brachii short head, BB, a flexor; and the long head of triceps brachii, TriLong, an extensor). The muscles were approximated with wires and were modeled with the Hill musculotendon model (Figure 1B and Figure 2A) [24], as described in [25].

**Figure 3:**
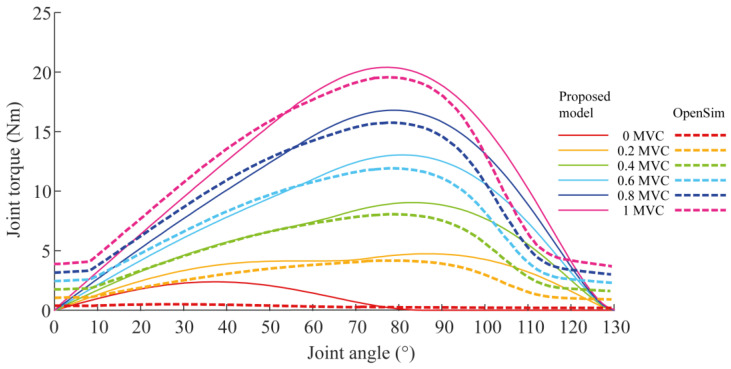
Comparison between the muscle torque–joint angle curves of the BRD muscle calculated with the model proposed in this study (continuous line) with respect to those calculated with the musculoskeletal modeling software OpenSim^®^ (dashed line) for different activation levels.

The model equations describing the behavior of each element of the Hill musculotendon model (Figure 2) are reported in Appendix A. The Hill model parameters were taken from Holzbaur [26]. However, these parameters were identified for a muscle model with a complex geometry, which cannot be approximated with a wire. In fact, the discrepancy could be ascribed to the different lengths that the muscles assume in a specific configuration if they are modeled with a wire, as in this study, or with a more complex geometry, as in Holzbaur. This led to a discrepancy in the optimal muscle-fiber length, the muscle-fiber-relaxation length, and the tendon-slack length. To overcome this issue, we scaled these muscle-specific values. The scaling parameter was chosen such that the peak force generated by the muscles simulated by both models occurred at the same joint angle. The gold-standard musculotendon model was the one implemented in the OpenSim^®^ project [27]. The scale factor and the scaled parameters that were used in this study are reported in the Appendix A, together with the stiffness of the parallel and the serial springs. The comparison between the joint torques exerted by the BRD muscle for different joint angles and muscle activations, as simulated with OpenSim^®^ and the model developed in this study with scaled parameters, is reported in Figure 3. 

#### 2.1.2. Model Range of Motion

Only upper-limb motion on the horizontal plane was considered. The elbow neutral position (angle 0°) was set when the forearm and the arm are aligned, and positive angles indicate elbow flexions. The shoulder neutral position (angle 0°) was set when the arm and the axis passing through the two shoulders are aligned, and positive shoulder angles indicate shoulder flexions.

**Figure 4:**
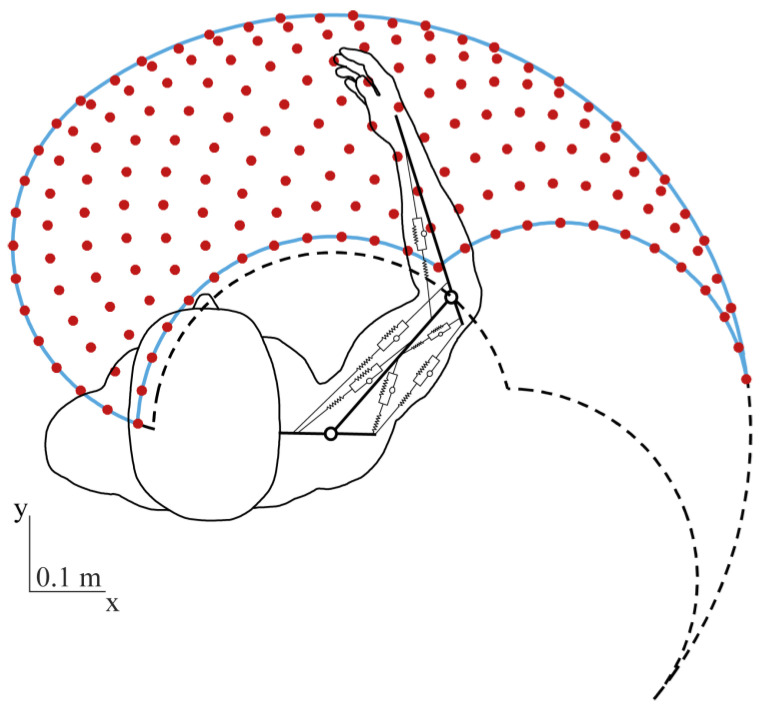
Workspace of the upper-limb model. The physiological workspace (black dashed line) and the workspace of the model (blue continuous line) are plotted. The positions in which the endpoint stiffness was calculated are indicated by red circles.

The physiological ranges of the motion of the elbow and shoulder joints are as follows [28]: [0°, 130°] and [−40°, –125°], respectively. However, the model cannot be used for the whole range of motion because of some intrinsic limitations. In fact, if one of the joints is completely extended, the modeled mono-articular muscles acting on it cannot exert any force because the moment arm is zero, while physiologically a force can be exerted also for joint angles close to the boundaries because of the physiological anatomy of the joints. Furthermore, the model cannot approximate the forces exerted by the shoulder joint for negative angles. Thus, the elbow and shoulder angles were restricted with respect to the values found in the literature. Both the elbow and shoulder joint angles’ ranges of motion were fixed between 5° and 125° and subdivided into 13 steps of 10°, resulting in a workspace of 169 distinct endpoint configurations (Figure 4). 

The activation of each muscle could assume a value between 0% and 100% (with a step of 10%) of the Maximum Voluntary Contraction (MVC). All the possible combinations of muscle activations (N = 1,771,561 = 11^6^) were tested with the limb placed at each of the 169 endpoint configurations.

#### 2.1.3. Equations for Endpoint Force Calculation 

**Figure 5:**
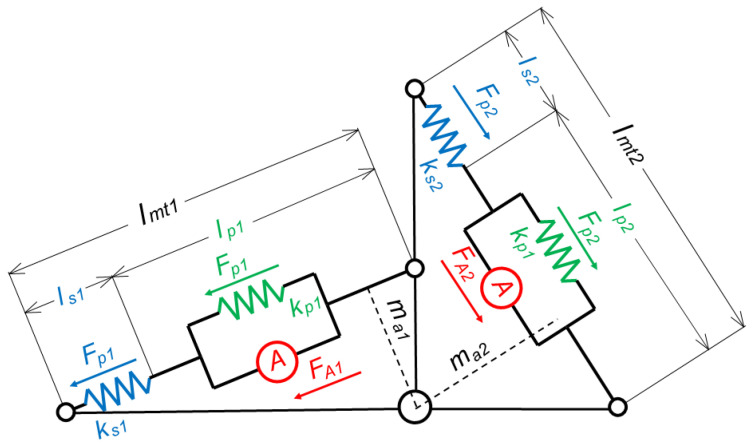
The forces exerted by two mono-articular muscles on a joint.

The force exerted by each muscle was calculated from the muscle–tendon length ($l_{m}$) that was estimated from the joint angles, the muscle attachment, and the activation (*m*), based on equations reported in Appendix A. The force-balancing equations for the mono-articular muscles acting on a joint (Figure 5) were the same as those described in previous studies [25,29], and they are also reported in Appendix A.

### 2.2. Experimental Paradigm

In this section, we describe the experimental paradigm implemented to validate the proposed approach.

#### 2.2.1. Participants

Eight right-handed subjects (age 23.8 (3.5), mean (std) across participants, five females) without any known neurological or musculoskeletal disease of the right upper limb, and with a normal or corrected to normal vision, participated in the experiment after giving written informed consent. The participants’ mean mass was 71 (4) kg, and the mean height was 1.7 (0.1) m. Since we did not expect any gender effect on the task performance or on the quality of the algorithm accuracy, we supposed that the participants’ sample reported in this study, even if not homogeneous or symmetric in terms of gender, would not alter the results. 

All procedures were conducted in conformity with the Declaration of Helsinki and were approved by the Ethical Review Board of the IRCCS Fondazione Santa Lucia.

#### 2.2.2. Experimental Setup 

The setup, which was already used in previous studies [20,30], is only briefly described here. For further information, please refer to the original studies.

**Figure 6:**
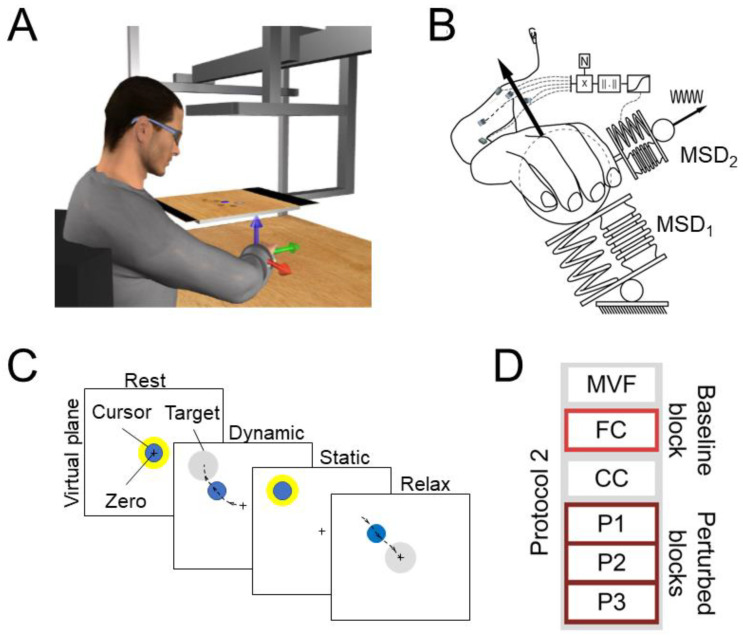
Experimental setup and protocol. (**A**) Setup. The reference system origin was centered in the participant’s palm. Arrows with different colors indicate the reference system axes (red: x-axis; green: y-axis; blue: z-axis). (**B**) Concept of the Perturbed blocks (P1, P2, and P3) of Protocol 2. The position of a virtual cursor was modeled as two connected mass-spring-damper systems. Participants controlled the mean position of the cursor by exerting a force on the first virtual mass-spring-damper system (MSD1) and controlled the cursor’s oscillation around the mean position by modulating the stiffness of a second virtual mass-spring-damper system (MSD2), which was perturbed by a constant sinusoidal force, through its level of co-contraction. The co-contraction was calculated as the norm of the projection of the instantaneous muscle activation along the null-space component of the EMG-to-force matrix, as calculated during the FC block. (**C**) Task. (**D**) The six blocks of which the protocol was composed were an initial MVF block, a baseline force control block (FC), a pure co-contraction block (CC), and three perturbed blocks (P1, P2, and P3). However, in this study, we used the only data collected during the FC (light red) and the P1, P2, and P3 (dark red) blocks.

Each participant sat in a chair in front of a desktop (Figure 6A), with their hand and forearm fixed in an orthosis that was rigidly connected to a 6-axis force and torque transducer (Delta F/T Sensor, ATI Industrial Automation, Apex, NC, USA) attached under the desktop. The participant’s hand was pronated, and the elbow was flexed at 90°, as measured with a goniometer. Participants wore 3D glasses and viewed a virtual scene displayed by a 3D 21-inch LCD monitor (Syncmaster 2233, Samsung Electronics Italia S.p.A., Cernusco sul Naviglio, MI, Italy) and reflected by a mirror placed halfway between the participant’s hand and the monitor. Real-time feedback of the exerted force was provided as the displacement of a virtual spherical cursor from a rest position. The motion of the cursor was simulated as a mass-spring-damper system under the force exerted by the participant (MSD1 in Figure 6B). 

#### 2.2.3. Experimental Protocol

The protocol, which is already presented in previous studies [20,30], is only briefly described here. For further information, please refer to the original studies.

The experiment was subdivided into 6 blocks (Figure 6D), each composed of several trials all performed in the same day. Each trial (Figure 6C) was composed of a rest phase, in which the subject was asked to keep the cursor in the rest position with a tolerance of 2% of the Maximum Voluntary Force (MVF), without applying any endpoint force and without contracting any muscle; a dynamic phase, in which the participant was asked to reach a displayed force target; and a static phase, in which the subject was asked to keep the cursor within the target. 

In the first MVF block, participants were asked to generate MVF along 20 different directions aligned with the vertices of a dodecahedron centered in the rest position. In the following baseline force control (FC) block, participants were asked to displace the cursor to reach one of the 20 targets positioned at the vertices of a dodecahedron, which was inscribed in a sphere centered at the origin and whose radius was 20% MVF. The cursor had to be maintained for 1 s within the target sphere, whose radius was 3% MVF larger than the radius of the cursor. Each target was presented 3 times, for a total of 60 trials. The third pure co-contraction (CC) block was composed of 15 trials in which participants were asked to co-contract their muscles to reduce the oscillation of the cursor around its mean position. The pure co-contraction block was introduced to familiarize subjects with the co-contraction task, and it was not analyzed in this study. In the last three perturbed blocks (P1–P3), participants were asked to reach and remain for 1 s within one of 20 targets, 3 repetitions each, positioned at the vertices of a dodecahedron inscribed in a sphere of 20% MVF radius and centered at the origin. Each target had a radius 3% MVF larger than the radius of the cursor. During these blocks, the cursor oscillated around its mean position as a mass-spring-damper (MSD2 in Figure 2B), perturbed by a disturbing force with a magnitude that increased across the different blocks. Participants could reduce this oscillation by raising the stiffness of MSD2 which was simulated in real-time according to the norm of the instantaneous projection of the muscle activation onto the null-space component of the EMG-to-force mapping, calculated online as the linear regression between EMG and force (“virtual stiffness”). For further details, please refer to [20]. The reduction of the amplitude of the cursor oscillation promoted the co-contraction of muscles.

Bipolar EMG signals were recorded with surface-active bipolar electrodes (Bagnoli system, Delsys Inc., Natick, MA, USA) from 12 muscles that were simulated in the upper-limb OpenSim model [31], which was implemented in the algorithm for the identification of the EMG-to-force mapping (see below). The investigated muscles were (1) brachioradialis, (2) biceps brachii long head, (3) biceps brachii short head, (4) pectoralis major, (5) anterior deltoid, (6) middle deltoid, (7) posterior deltoid, (8) triceps brachii lateral head, (9) triceps brachii long head, (10) infraspinatus, (11) teres major, and (12) latissimus dorsi. 

EMG activity was acquired at 1000 Hz, bandpass filtered (20–450 Hz), and amplified with a 1000 gain. Subjects’ skin was cleansed with alcohol, and electrodes were placed based on recommendations from SENIAM [32] and by palpating muscles to locate the muscle belly and orienting the electrodes along the main direction of the fibers [33]. An analog-to-digital PCI board (PCI-6229; National Instruments, Austin, TX, USA) digitalized, at 1 kHz, the EMG and force data. Data acquisition, experiment control, and data analysis were made with custom software written in MATLAB^®^ (MathWorks Inc., Natick, MA, USA) and Java.

The EMG data were processed with an initial subtraction of the mean value to remove any offset. Data were then rectified and low-pass filtered (2nd order Butterworth with 1 Hz cutoff frequency). The mean activity collected during the rest phase was subtracted from the rest of the data; the EMG activities of each muscle were normalized to the maximum value collected during the MVF block (i.e., initial block in Figure 6D) and finally resampled at 100 Hz to reduce the computational cost. Force data were low-pass filtered (2nd order Butterworth with a 1 Hz cutoff) and resampled at 100 Hz. Both the EMG and force data that were collected during the static phase of each trial, i.e., during the 1 s in which participants were required to keep the cursor within the target, were averaged (Figure 6C). 

#### 2.2.4. EMG-to-Force Matrix Estimation

A matrix, *H*, with dimensions [force dimensions × number of muscles], was used to linearly approximate [34,35] the mapping of EMG signals, ***m***, with dimensions [number of muscles × number of samples] into the endpoint force, ***f***, with dimensions [force dimensions × number of samples]:

(1) f=H·m

The EMG-to-force matrix, *H*, that was first calculated from data simulated though the musculoskeletal model, was obtained as the regression of the muscle activation onto the endpoint force, using the MATLAB function *regress*, as proposed in the literature [9,36,37]. Since the endpoint of the musculoskeletal model changed with the posture, a different *H* matrix was calculated for each configuration. Moreover, different matrices were calculated from different sets of muscle activations and endpoint forces depending on the simulated muscle activations. In particular, each set was composed of those muscle activations in which any muscle had an activation equal to or lower than *i*, where *i* = [10% 100%] (step 10%) of the MVC, and therefore, ten different *H* matrices were calculated. For example, the *H* matrix calculated with muscle activations equal or lower than 30% MVC, and it also contains data used for the calculation of the *H* matrix calculated with muscle activations equal to or lower than 10% MVC and 20% MVC. 

**Figure 7:**
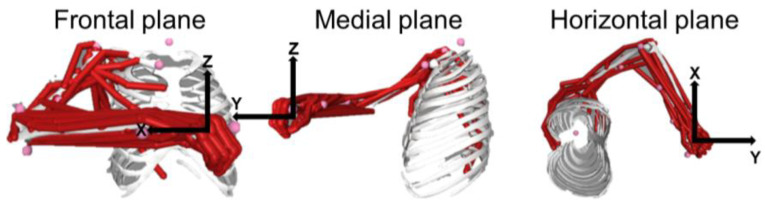
Musculoskeletal model posture on a frontal (**left panel**), medial (**middle panel**), and horizontal (**right panel**) planes. The model was set to assume the same posture that participants assumed during the isometric task.

The *H* matrix was also calculated from experimentally collected data, using a musculoskeletal model (i.e., OpenSim^®^ project [27], version 4.3). The approach was previously described in [30], and it is only briefly reported here. Firstly, the joints of the OpenSim MoBL-ARMS model [31] are set in a posture that is similar to the one assumed by participants during the isometric experiment (shoulder flexion–extension angle, 55°; shoulder abduction–adduction angle, 65°; shoulder rotation angle, 60°; elbow flexion angle, 90°; wrist prono-supination, 30°; wrist deviation, 0°; and wrist flexion, 0° (see Figure 7)).

The scaling of the OpenSim musculoskeletal model was then performed with the OpenSim’s scaling tool, which allowed us to scale the mass of the model, the segments’ length, and the maximum isometric force of each of the model muscles according to the participant’s total mass and height. Then the moment arms of each of the considered muscles, with respect to each joint, were calculated with the OpenSim’s Inverse Kinematic tool. The OpenSim Inverse Dynamic tool allowed for the calculation of the inverse Jacobian by determining the torque exerted, in isometric conditions, at each joint, after applying seven external simulated forces (0 N, ±0.1 N, ±0.2 N and ±0.3 N) at the level of the wrist along each axis. The components of the inverse Jacobian were determined by calculating the linear-regression slope of each of the simulated forces onto the corresponding torques. Finally, the EMG-to-force mapping was estimated as follows:

(2) $$H_{experimental}=J^{-1}\cdot M\cdot F_{MAX}$$

where $F_{MAX}$ maps muscle activations onto the tension that they exert, *M* maps muscle tensions onto the torques generated at each joint, and $J^{-1}$ maps the joint torques onto the force exerted at the endpoint.

### 2.3. Endpoint Stiffness Calculation

Endpoint stiffness is usually estimated by displacing the endpoint and measuring the restoring force [1]. In this study, the displacement was applied to the joints and not to the endpoint. The angular deflection applied to the joints was assumed to be small enough to justify the approximation of the angle–muscle torque curve with a linear relation [38]. This approximation may lead to a discrepancy with the literature that is expected to have only a negligible influence on the calculation of the endpoint stiffness, but it leads to a significant reduction of the calculation time.

The endpoint stiffness, *K*, was calculated as the ratio between the endpoint force variation, ΔF, with respect to the endpoint displacement, Δε:

(3) K=ΔFΔε

The force variation, ΔF, was calculated as the difference between the endpoint force exerted after and before the displacement. When perturbations were applied, the endpoint stiffness matrix was (almost) symmetrical [39]. Therefore, as usually found in the literature [5], the endpoint stiffness in the horizontal plane was represented as an ellipse and in the 3D space as an ellipsoid. The distance of each point from the ellipse or ellipsoid center indicates the force recorded after an endpoint displacement. 

The equation of the stiffness ellipse centered in the origin is as follows:

(4) x12a2+y12b2=1

where *a* and *b* are the projections along the principal axes $x_{1}$ and $y_{1}$, defined as follows:

(5) $$\left(\begin{array}{c} x_{1}\\ y_{1} \end{array}\right)=\left(\begin{array}{cc} \cos\alpha & \sin\alpha \\ -\sin\alpha & \cos\alpha \end{array}\right)\cdot \left(\begin{array}{c} x\\ y \end{array}\right)$$

where α represents the rotation angle of the major axis. 

The equation of the stiffness ellipsoid centered in the origin is as follows:

(6) x12a2+y12b2+z12c2=1.

where *a*, *b*, and *c* are the projections along the principal axes $x_{1}$, $y_{1}$, and $z_{1}$, which are defined as follows:

(7) $$\left(\begin{array}{c} x_{1}\\ y_{1}\\ z_{1} \end{array}\right)=R_{z}R_{y}R_{x}\cdot \left(\begin{array}{c} x\\ y\\ z \end{array}\right)$$

where $R_{x}=\left(\begin{array}{ccc} 1 & 0 & 0\\ 0 & \cos\alpha & -\sin\alpha \\ 0 & \sin\alpha & \cos\alpha \end{array}\right)$, $R_{x}=\left(\begin{array}{ccc} \cos\beta & 0 & \sin\beta \\ 0 & 1 & 0\\ -\sin\beta & 0 & \cos\beta \end{array}\right)$, and $R_{x}=\left(\begin{array}{ccc} \cos\gamma & -\sin\gamma & 0\\ \sin\gamma & \cos\gamma & 0\\ 0 & 0 & 1 \end{array}\right)$ are, respectively, the matrices describing the rotations of the major, middle, and minor axes around the Cartesian axes *x*, *y*, and *z* of the *α*, *β*, and *γ* angles.

The musculoskeletal model implemented in this study could only exert forces and be displaced along the horizontal plane; therefore, the endpoint simulated stiffness is represented as an ellipse. In contrast, as experimental data were collected during the exertion of tri-dimensional forces, the endpoint experimental stiffness is represented as an ellipsoid.

#### 2.3.1. Estimation of the Simulated Stiffness

In the model, since the variation of the force exerted by the passive elements was negligible with respect to the one exerted by active elements, the force variation (ΔF) was calculated as the difference between the force generated only by the active elements of the muscles, both after and before the displacement. The rotated ellipse centered in the origin was univocally defined by three points that identified the major and the minor axes and their rotation with respect to the reference system. Therefore, three endpoint displacements were applied to calculate the stiffness ellipse parameters, during which muscle activations were kept constant. The displacements were defined as three different combinations of elbow and shoulder angular deflections: (1) 0.01° deflection applied to the elbow joint, 0.01° deflection applied to the shoulder joint; (2) −0.01° deflection applied to the elbow joint, −0.01° deflection applied to the shoulder joint; and (3) 0.01° deflection applied to the elbow joint, −0.01° deflection applied to the shoulder joint. The stiffness ellipse was calculated for each of the 169 upper-limb postures and each of the 1,771,561 muscle activations.

#### 2.3.2. Estimation of the Experimental Stiffness 

We simulated 14 endpoint displacements through the OpenSim musculoskeletal model [31]. The first 8 perturbed postures were determined by separately deflecting a single joint by +1° or −1°. The deflected joints were the shoulder flexion–extension, the shoulder abduction–adduction, the shoulder rotation, and the elbow flexion angles. The other 6 perturbed postures were determined by simultaneously deflecting two joints, both by +1° or −1°. The two joints that were simultaneously deflected were the shoulder flexion–extension and the shoulder abduction–adduction, the shoulder rotation and the elbow flexion, and the shoulder abduction–adduction and the elbow flexion angles. The endpoint force variation was identified by multiplying the experimental EMG activation by the EMG-to-force mapping, as calculated for each different posture with the procedure described in Section 2.2.4, and subtracting the product between the experimental EMG activation by the EMG-to-force mapping calculated at the original posture. An optimization determined the ellipsoid that best fit the stiffnesses calculated after the 14 displacements.

### 2.4. The Norm of the Null-Space Component of Muscle Activation as an Approximation of the Endpoint Stiffness

In this section, we described the novel approach to approximate the endpoint stiffness, using the norm of the null-space component of the muscle activations.

#### 2.4.1. Implications of Muscle Redundancy

The forces exerted by a human operator can be represented in a vector space (i.e., the force space) whose axes represent the force exerted along different space dimensions. Similarly, the muscle activations could also be represented as a vector space (i.e., the muscle space) whose axes represent the activations of all collected muscles. Thus, the dimensionality of the muscle space is equal to the number of muscles, and each vector represents the set of muscle activations recorded at a single time sample. If the force space has a lower dimensionality with respect to the muscle space, we can project any muscle activation, *m*, along two orthogonal subspaces defined by the EMG-to-force matrix [22]: the row space, whose elements represents the muscle activation vector with the minimum norm that generates a given endpoint force; and the null space, whose elements are muscle activation vectors that map onto zero vectors of the force space. Thus, the null-space projection of a muscle activation vector represents the component of muscle activation vector that does not generate any endpoint force.

**Figure 8:**
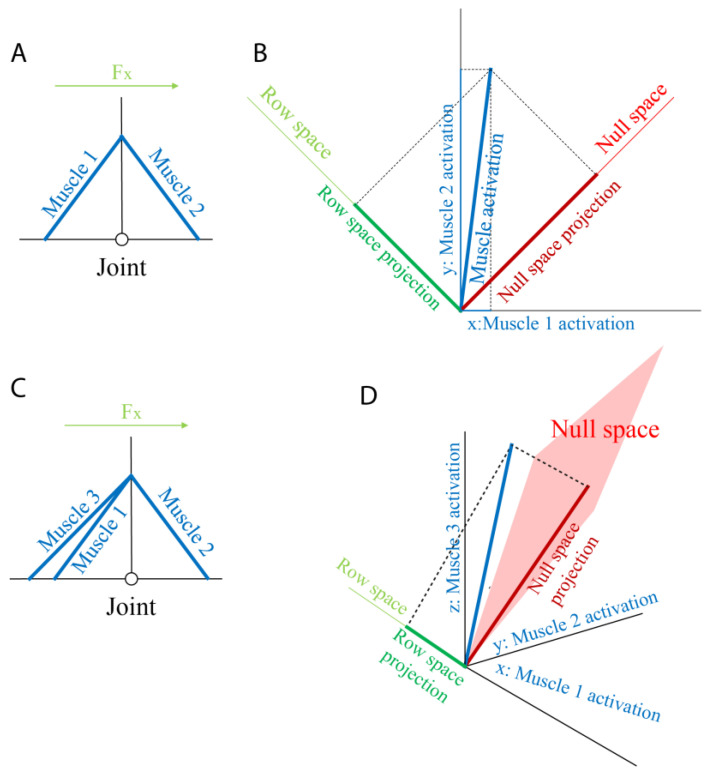
Example of the identification of the projection of the muscle activation onto the row and the null spaces. (**A**) A model made of one joint and two identical antagonist muscles (Muscle 1 and Muscle 2, blue lines). The positive direction of the force is represented as a green arrow (Fx). (**B**) The two-dimensional muscle space whose axes represent the activations of the two muscles (Muscle 1 and Muscle 2 on the x- and y-axis, respectively). The null (red line) and row (green line) one-dimensional subspaces are identified. An example of a muscle activation ([0.1 0.8], blue), together with its projections on the null (dark red) and the row (dark green) spaces, is reported. (**C**) A model comprising one joint and three muscles. Two muscles (Muscle 1 and Muscle 2, blue lines) are antagonist to a third one (Muscle 3). The positive direction of the force is represented by a green arrow (Fx). (**D**) The three-dimensional muscle space whose axes represent the activations of the three muscles (Muscle 1 on the x-axis, Muscle 2 on the y-axis, and Muscle 3 on the z-axis). The null (red plane) and row (green line) subspaces are identified. An example of a muscle activation ([0.1 0.4 0.8], blue), together with its projections on the null (dark red) and the row (dark green) spaces, is reported.

The identification of the null and row spaces of the *H* matrix can be easily performed on a model with a single joint and two antagonist muscles. If, for example, we assume that the joint is flexed at 90° (see Figure 8A), the *H* matrix is as follows:

$$H=\left[\begin{array}{cc} -1 & 1\\ 0 & 0 \end{array}\right]$$

The null space, *N*, computed with the MATLAB^®^ function *null*, and the row space, $H^{+}$, computed as the pseudo-inverse of the *H* matrix with the MATLAB function *pinv* (in this case, only one of the two components is considered, because the other is [0 0]^T^), are as follows:

$$N=[\begin{array}{c} 0.71\\ 0.71 \end{array}],\;H^{+}=[\begin{array}{c} -0.71\\ 0.71 \end{array}].$$

Since the muscle space is two-dimensional and the row space is one-dimensional, the null space, whose dimension is the difference between the muscle-space and the row-space dimensions, is one-dimensional too, and by definition, *N* and $H^{+}$ are orthogonal (Figure 8B), and *N* represents the simultaneous and equal activation of both muscles. A modulation of the muscle activation only along the null space leads to the modulation of the endpoint stiffness with a fixed endpoint force. In this case, then, the null-space projection of the muscle activation is strictly related to the stiffness, and it is coincident with the muscle subspace that generates only stiffness (i.e., the stiffness space). The projection of a muscle activation vector onto the row space leads to a muscle activation in which at least one component is negative. Since the non-negativity is a physiological constraint, we can conclude that any muscle activation—except for in the case in which no muscles are activated—has a component of null space that is required to satisfy the non-negativity constraint. This observation is true also if only one of the two antagonistic muscles is activated. Therefore, any physiological activation leads to a modulation of the null space, with a consequent modulation of the stiffness, as noticed in the literature [40].

In the case reported in Figure 8C, if the two antagonist muscles are parallel, the muscle space becomes three-dimensional, and the *H* matrix becomes as follows:

$$H=[\begin{array}{ccc} -1 & -1 & 1\\ 0 & 0 & 0 \end{array}].$$

The force space remains one-dimensional because the force could be exerted along only one axis, and the force vector becomes as follows:

$$H^{+}=[\begin{array}{c} -0.58\\ -0.58\\ 0.58 \end{array}].$$

The dimensionality of the null space grows from 1 to 2, and its components are as follows:

$$n=[\begin{array}{cc} -0.58 & 0.58\\ 0.79 & 0.21\\ 0.21 & 0.79 \end{array}].$$

However, since only stiffness modulation around the single joint is feasible, the stiffness space is still one-dimensional. Therefore, we could conclude that the null space is the sum of a component that generates the stiffness (stiffness space) and a component that generates neither the stiffness nor the force.

Despite this, the stiffness space—or in this, case the stiffness vector—cannot be easily discriminated in the muscle space and the null space, and consequently the projection of the muscle activation onto the null space can be easily determined based on the *H* matrix. 

#### 2.4.2. The Projection of the Muscle Activation Vector onto the Null Space 

The muscle-space dimension of the implemented simulation had 6 dimensions or degrees-of-freedom (DOFs), because 6 muscles were modeled, and the muscle-space dimension of the experimental paradigm was 12. In the simulated model, the shoulder was abducted at 90°, and the arm-limb motion and the forces that the model could exert were only along the horizontal plane; then the force space had 2 DOFs. In contrast, the force exerted during the experimental paradigm spanned the whole tri-dimensional space, and, therefore, the force space had 3 DOFs. Consequently, the null space of the model had 4 DOFs (6 muscle-space DOFs—2 force-space DOFs), 3 of which were the dimension of the stiffness space, and the dimension of the component of null space that did not exert any stiffness was 1 (4 null-space DOFs—3 stiffness DOFs), while the null space of the experimental paradigm had 9 DOF (12 muscle space DOFs—3 force-space DOFs), 3 of which were the dimension of the stiffness space; and 6 DOFs did not exert any stiffness.

The component of the muscle activation, *n*, that did not generate any endpoint force was calculated by projecting the muscle activation, *m*, onto the null space, *N*:

(8) $$n=N^{T}\cdot N\cdot m$$

The component that lay on the null space was calculated for each muscle activation.

### 2.5. Statistics 

The quality of the reconstruction of the stiffness ellipse or ellipsoid axes as a linear function of the projection of the muscle activation onto the null space was tested. We also investigated the relation between the norm of the null space of the muscle activation with the endpoint stiffness ellipse area (i.e., the product of the major and the minor axes, scaled by π) or the endpoint stiffness ellipsoid volume (i.e., the product between all axes, scaled by 4π/3), as proposed in previous works [38].

The relationship between the ellipse or ellipsoid axes and the norm of the null-space projection of the muscle activation was assessed through a regression analysis (MATLAB function *regress*). A significant relation was determined through the *p*-value (threshold, *p* < 0.05).

The quality of the reconstruction of the endpoint stiffness axes or area/volume through the null-space component of the muscle activation was assessed through the Variance Accounted For (VAF):

(9) $$\mathrm{VAF}=1-\frac{\sum_{i}{\left(X_{i}-\tilde{X}\right)}^{2}}{\sum_{i}{\left(X_{i}\right)}^{2}}.$$

where $X_{i}$ is the *i*-th amplitude of one stiffness axis or the area/volume of the stiffness ellipse/ellipsoid, and $\tilde{X}$ is the implemented model, i.e., the norm of the null-space component of the muscle activation. To reject the hypothesis that the same quality of the fitting was obtained by chance, we randomly shuffled the norm of the null-space muscle component, and then we calculated the VAF again with the axes or the area/volume of the stiffness ellipse/ellipsoid. This approach disrupted the modulation of the data that the model could fit, without affecting the mean value. The 95th percentile, over 500 random shuffling, was retained. In the analysis on experimental data, a paired Student’s *t*-test was used to test the null hypothesis that the VAF calculated from experimental data and the 95th percentile of the VAF obtained from random shuffling the norm of the null space of muscle activation, calculated on data collected from different participants during different blocks, came from the same distribution. A *p*-value lower than 0.05 rejected the null hypothesis.

In the musculoskeletal simulation, the regression and the fitting were separately performed on each endpoint displacement on a subset of simulations in which all the muscles had activations lower than or equal to the threshold, *i*, where *i* = 10, 20, 30, 40, 50, 60, 70, 80, 90, or 100% of the MVC.

In the experimental paradigm, the regression and the fitting were performed separately on data collected from each participant during the baseline or the perturbed blocks. Moreover, a paired Student’s *t*-test was used to assess whether data collected during different blocks showed different reconstruction levels (threshold, *p* < 0.05).

## 3. Results

### 3.1. Musculoskeletal Model 

**Figure 9:**
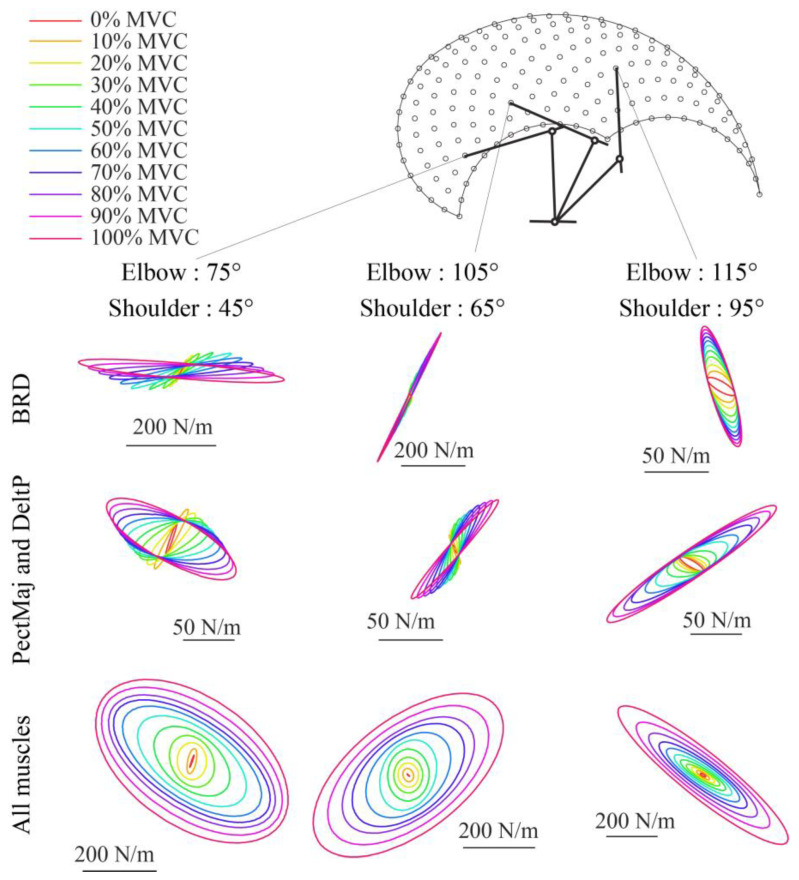
Examples of endpoint stiffness ellipses calculated for different arm configurations and muscle activations from the musculotendon simulation. In the first row, only the BRD muscle is activated at different levels, while all the other muscles have a zero activation. In the second row, both the PectMaj and the DeltP are activated with the same value, while all the other muscles have a zero activation. In the third row, all the muscles are activated with the same activation. Different values of activations are coded with different colors.

The shapes, axes directions, and axes amplitudes that were simulated with the musculoskeletal model were consistent with those reported in the literature [41,42] (see Figure 9). 

**Figure 10:**
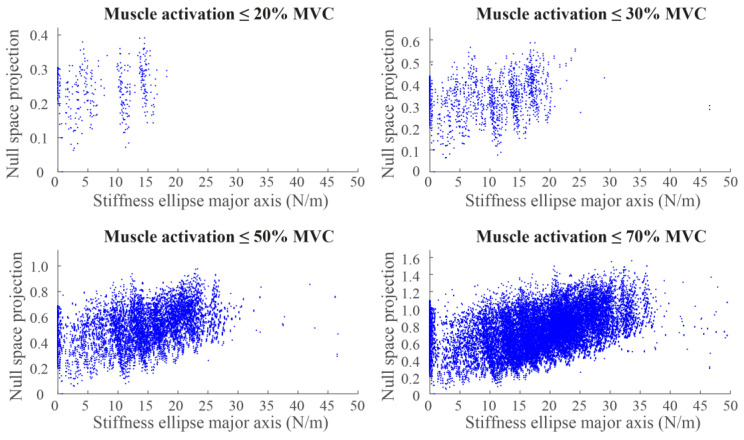
Examples of the relation between the major axis of the stiffness ellipse (x-axis) and the norm of the null-space projection of the muscle activations (y-axis) calculated on different sets of muscle activations simulated in a specific endpoint pose.

In Table 1, the percentage of the 169 poses that showed a significant positive regression (*p* < 0.05) between the norm of the null-space component of the muscle activation and the ellipse axes and area is reported. To investigate the effect of the muscle-activation amplitude, separate regressions were performed on data subsets. The muscle patterns that composed each subset showed activations of all muscles lower than or equal to *i*, where *i* = 10, 20, 30, 40, 50, 60, 70, 80, 90, or 100% of the MVC. The major axis, the minor axis, and the area all showed a significant regression with the norm of the null-space projection of muscle activations in most poses (>50%) in all the subsets of muscle patterns, except for the one whose maximum activation was 10% of the MVC. The fraction of poses showing a significant relation with the major axis was >90% if the subset included muscle activations >30%, and a significant relation with the minor axis and area was >90% if the subset included muscle activations >40%. In Figure 10, examples of the relation between the major axis of the stiffness ellipse and the norm of the null space of the muscle activation calculated at a specific pose and for different subsets of muscle patterns are reported.

The quality of the reconstruction, which was tested with the VAF, showed that the norm of the null-space component of the muscle activation provided a good fitting (VAF > 70% MVC; see Table 1) that was higher than the one obtained by chance of the only major axis of the stiffness ellipse for all the muscle activations’ patterns. In contrast, the fitting of the minor axis was lower than the one obtained by chance for all muscle activations, except when high muscle activations (>70% MVC) were allowed, while the fitting of the area was always lower than the one obtained by chance. Moreover, the fitting of both the minor axis and the area showed a poor reconstruction (VAF < 50% [43]). 

### 3.2. Experimental Paradigm 

**Figure 11:**
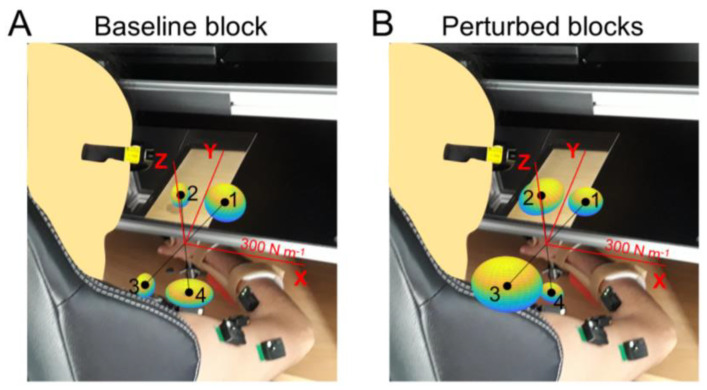
Examples of endpoint stiffness ellipsoids calculated along four force targets lying on the horizontal *x*/*y* plane that were collected during the baseline block (**A**) or perturbed blocks (**B**). The stiffness ellipsoids are translated in order to make the ellipsoid center and the target position along which the ellipsoids were calculated, i.e., the black dots, coincident. Numbers close to the target positions refer to the target index. Red lines indicate the reference system, which is centered on the palm when participants are not exerting any endpoint force, and they all have the same length in the space (300 N∙m^−1^).

An example of the stiffness ellipses calculated from data collected during a baseline and perturbed blocks when a participant was exerting forces along four directions is reported in Figure 11. 

In line with the results obtained with the simulated data, the data collected during the baseline block from all participants showed a significant relation (*p* < 0.05, reported in Table 2 with an asterisk) between the norm of the null-space component of the muscle activation with the major, middle, and minor axes, and seven out of eight participants also showed a significant relation with the stiffness ellipsoid volume. In contrast, while all participants showed a significant relation of the norm of the null-space component with respect to the middle and minor axes, 2 participants (ID 4 and 8) showed no significant relation with the major axis, and 1 participant (ID 7) showed no significant relation with the ellipsoid volume.

A Student’s *t*-test identified different fitting accuracies in the reconstruction of data collected from the baseline or the perturbed blocks (see Table 2), both testing one of the axes or the ellipsoid volume. In particular, the null-space component of the muscle activation better reconstructed all the principal axes (*p*: 0.001, 0.001, and 0.005 for the major, middle, and minor axes, respectively) and the volume (*p*: 0.002) of the ellipsoids calculated from data collected during the perturbed blocks with respect to the baseline. 

However, a significant difference in the VAF between the fitting of the data with the norm of the null-space component of muscle activation, as calculated from the data, and as the 95% over 500 random shuffling, identified a significant fitting of the only major axis in data collected during the baseline block, or the major and the middle axes in data collected during the perturbed blocks (see last column of Table 2). Moreover, while all participants showed a good fitting of the major and middle axes (VAF > 70%) of the ellipsoids calculated from data collected during the perturbed block, only two participants showed a good reconstruction of the major axis calculated form data collected during the baseline block. A good fit of the minor axis of the ellipsoid was identified in six participants (while the other two participants showed a VAF >65%), and only four participants showed a good reconstruction. No participants showed a good reconstruction of the volume of the stiffness ellipsoid calculated during the perturbed blocks, while two participants showed a good fitting of the ellipsoid volume calculated from data collected during the baseline block.

## 4. Discussion

The real-time estimation of the upper-limb stiffness could be useful in many clinical and industrial (ergonomy) applications. In fact, stiffness is physiologically modulated by participants to reduce the effects of an external perturbation [44] and to improve movement accuracy [45], while non-physiological stiffness modulation can be observed in stroke patients [46]. Therefore, different approaches have been proposed in the literature to estimate, in real-time, the endpoint stiffness modulation due to muscle activation. A short-range stiffness estimation, which was based on the geometry and the active forces exerted by all the recruited muscles [47], could account for 91% of the variance in stiffness shape and 82% of the variance in stiffness area [38], while EMG-driven approaches, based on complex musculoskeletal models, could fit the stiffness of the knee with an average 93% accuracy [48]. However, information on the muscles’ characteristics may not be easily accessible, and musculoskeletal models may require customized solutions [17]. Therefore, this study presented an approach that requires a low computational cost; does not need for any foreknown characteristics of the muscles; and can be used with healthy participants, neurological patients, or amputees, since it does not assume any specific musculoskeletal geometry. This approach relies on the norm of the projection of the muscle activations onto the null space of the EMG-to-force mapping, i.e., the component of muscle activation that does not generate any endpoint force, as a linear approximation of the endpoint stiffness. Therefore, this approach accounts for the redundancy of the musculoskeletal system by estimating the stiffness exerted by several muscles acting on different joints.

Two validations were performed: one on simulated data, using an upper-limb model composed of two joints (elbow and shoulder joints) and six muscles (two antagonist monoarticular muscles acting on the elbow, two antagonist monoarticular muscles acting on the shoulder, and two antagonist biarticular muscles), and the other on experimental data collected from twelve upper-limb muscles during a task requiring the exertion of three-dimensional isometric forces, both with and without the explicit requirement of co-contraction. A significant linear relation was demonstrated between the norm of the null space of the muscle activations, with respect to the ellipse axes or area, in most of the poses, but only if the maximum muscle activations were high enough (a significant relation with the major or minor axes occurred in >90% of the model poses if the muscle patterns were activated up to, respectively, >30% MVC or >40% MVC). Similarly, experimental data collected during the baseline block, i.e., without an explicit modulation of the endpoint stiffness, identified a significant relation between the norm of the null space of muscle activations and the ellipsoid axes in all participants, and a significant relation with the ellipsoid volume in seven out of eight participants. Experimental data collected during the perturbed block, i.e., which required an explicit modulation of the endpoint stiffness, identified a significant relation between the norm of the null space of muscle activations and the middle and minimum ellipsoid axes in all participants, a significant relation with the maximum ellipsoid axis in six out of eight participants, and with the ellipsoid volume in seven out of eight participants. 

In the literature, an EMG-driven approach, determining the stiffness of the knee after the activation of seven lower-limb muscles, led to a fitting with a VAF of 84 ± 5% if trials required low co-contraction levels, which dropped to 80 ± 7% when the required co-contraction was higher [49]. A recent approach, called the Short Segment-Structural Decomposition SubSpace, achieved a VAF of 87% in the estimation of the knee stiffness [50]. We also assessed the fitting accuracy through the VAF, but its statistical significance was tested by calculating the VAF after randomly shuffling the norm of the null-space projection of the muscle activation and by retaining the 95th percentile as a significance threshold over 500 repetitions. The proposed statistics were conservative since they disrupted the common variations between the two signals without affecting their mean values. The data simulated through the musculoskeletal model demonstrated that the norm of the null-space projection of the muscle activation could only approximate the major axis of the stiffness ellipse with an accuracy (>70%) that was higher than the one achieved by chance. In contrast, despite the fact that the fitting of the major ellipsoid axis calculated during the baseline block of the experimental protocol was significantly higher than a random distribution (*p*: 0.019), its accuracy was quite low (mean (std) across participants: 60 (14)%), but the quality achieved in the fitting of the major and middle axes during perturbed blocks (VAF 82 (5)% both axes; *p* < 0.001 for the fitting of the major axis, and *p* = 0.006 for the fitting of the middle axis) was in line with other similar approaches [49,50], even if lower than the one achieved with more complex approaches [38,48]. Therefore, our results suggest that the norm of the null-space projection of muscle activation is a valid approximation of the major axis of a stiffness ellipse and of the major and middle axes of a stiffness ellipsoid, but only for higher muscle activations or when a voluntary co-contraction is exerted.

The relation between the fitting accuracy and the muscle-activation amplitude may be a consequence of the design of the proposed approach, which is sensible not only to the component of the null space that modulates the endpoint stiffness but also to the component that does not affect the force or stiffness. If no explicit co-contraction is required by the task, i.e., during the baseline block of the experimental protocol or when low muscle activations are simulated in the musculoskeletal model, the null-space component that modulates neither the force nor stiffness would be predominant, and the fitting accuracy would be affected. On the contrary, if voluntary co-contractions are required by the task, the component of the muscle activation which modulates the endpoint stiffness would be prevalent, and the algorithm would return a valid approximation. The physiological meaning of the null-space component that does not modulate either the force or the stiffness is still unknown, but it could be a consequence of the non-negativity of the muscle activations [20]. Despite the fact that it was recently demonstrated that healthy participants are able to voluntarily modulate the null-space components of the muscle activation that were recruited during a co-contraction task [51], the voluntary modulation of the null-space components that were not involved in a co-contraction will need to be investigated in future studies.

The estimation of the stiffness as the norm of the null-space component of muscle activation may be influenced by the quality of the EMG-to-force reconstruction. Different approaches for the estimation of the EMG-to-force mapping have been proposed in the literature. During isometric tasks, in which the non-linearities due to muscle contraction velocity and muscle length can be neglected, an approximation of the EMG-to-force relation via linear mapping, as performed in this study, may be acceptable [52,53,54], and it may be eventually improved by introducing foreknown anatomical constraints [13,30,55,56,57]. Moreover, an interesting recent study [58] proposed an innovative approach for the rapid estimation of the joint torque or velocity from the muscle activations. This approach is based on the proportion between the muscle activation and the exerted force, and it relies on the Hill musculoskeletal model and on a further optimization that is aimed at balancing the forces generated by the muscles acting on each joint. Since the approach proposed in this study could be applied independently from the algorithm selected to determine the mapping between the muscle activation and the endpoint force, this study did not aim at contributing to the debate on the selection of the best algorithm for the EMG-to-force processing. However, better-fitting accuracies of the endpoint stiffness are expected if a better estimation of the EMG-to-force mapping is available. Therefore, further studies would test the reconstruction quality that can be achieved through different approaches for the estimation of the EMG-to-force mapping. 

Despite the fact that the proposed approach was tested under isometric conditions, it could be expanded to dynamic tasks by calculating the time-varying null space—as already proposed in the literature to control redundant robots [59]—of a time-varying EMG-to-force mapping. The effect of the joint angle on the EMG-to-force mapping could be determined by interpolating the EMG-to-force matrices calculated on different postures [37] or through more complex approaches based on multidimensional B-splines [60]. Therefore, despite the fact that the approach described in this study did not allow us to determine the endpoint position or exerted force, a proper estimation of the EMG-to-force processor would also provide an estimation of the system’s kinematic or dynamic, and its combination with our approach would then also provide an estimation of the exerted stiffness. Moreover, since the linear approximation of the EMG-to-force mapping was demonstrated to be consistent across different days [36,61], future works will test whether the estimation of the stiffness characteristics as the null-space component of the muscle activation is also consistent across sessions. 

### 4.1. Limitations

The proposed method also has some limitations. In particular, it approximates the endpoint stiffness as an isotropic function, and therefore, different from other approaches proposed in the literature [38], the directions of the principal axes of the stiffness ellipse cannot be determined. Despite the fact that the axes’ direction of the stiffness ellipse, as identified during an isometric task, displayed small changes even if participants were provided with real-time visual feedback [62], a tuning of the stiffness was identified during dynamic tasks [63]. However, despite this limitation, the proposed approach is particularly promising as the control law of the stiffness exerted by wearable robotic devices with variable stiffness, such as exoskeletons or prostheses, since the control logic of the intervention would be proportional to the level of limb stiffening that the operator aims to generate, despite its shape. 

The stiffness of a limb does not only depend on the actual co-contraction of muscles, but also on their mechanical properties—for which some proteins are responsible [64,65]—and on the elicited stretch reflex [66,67]. These two components cannot be estimated through our approach, which, as with all the other approaches based on muscle activity signals, can only discern the active voluntary modulation of the stiffness centrally controlled by the modulation of the muscle activation. Therefore, our approach could only determine the stiffness that a human operator voluntarily exerts, but not the mechanical characteristics of the system, which require more complex musculoskeletal systems. 

Despite the fact that the proposed approach did not require the knowledge of the posture assumed by participants, a discrepancy in the calculation of the posture assumed during the experimental protocol may lead to errors in the definition of the OpenSim model which propagate in the calculation of the stiffness ellipses, with a consequent reduction in the fitting accuracy. To overcome this issue, future studies aiming to test this algorithm, or studies involving a dynamic task, should measure the participant posture through a motion-capture system.

### 4.2. Future Developments

The implemented algorithm may be particularly useful for a human-in-the-loop control, where the experimental apparatus, or the myo-controlled robotic device, is already designed to estimate the mapping between EMG and exerted force [13]. Since only data collection from EMG electrodes is required, the proposed approach could be a valid solution for the real-time estimation of the endpoint stiffness that the human operator intends to exert to control wearable robotic devices.

Further studies will experimentally investigate the controllability of the stiffness of a wearable robotic device, through the norm of the null-space component of the operator muscle activation, both during an isometric force exertion task and during a dynamic task. 

The proposed approach could also be implemented in rehabilitation therapies to estimate the pathological co-contraction, e.g., in post-stroke [68,69] or dystonic [70,71] patients. A simple measurement of the level of pathological co-contraction would provide useful information on how efficiently a physical therapy intervention is proceeding. An estimation of the pathological co-contraction level may also be provided to a human therapist during a tele-rehabilitation paradigm [72,73] or to a robotic device during a robotic-rehabilitation paradigm [74,75,76] in order to define the assistance level.

## 5. Conclusions

In conclusion, this study proposed an approach for the estimation of the endpoint upper-limb stiffness from the component of the muscle-activation vector projected onto the null space of the EMG-to-force mapping, i.e., the component of the muscle activation that does not generate any endpoint force. This approach was validated with a musculoskeletal model with two joints and three pairs of antagonist muscles, and with experimental data, and could easily be implemented in wearable robotic applications. 

## Figures and Tables

**Table 1:** Quality of the fitting of the major axis, the minor axis, or the area of the stiffness ellipse, as simulated with the musculoskeletal model, as the product of the null-space projection of the muscle activation normalized to the ratio between the mean null-space projection of the muscle activation divided by the mean value of the ellipse axes or area. The top rows show the percentage of the musculoskeletal model poses, showing a significant (*p* < 0.05) regression between the stiffness ellipse axes or area and the norm of the null-space projection. The bottom rows show the VAF of the approximation of the stiffness ellipse axes or area as the product of the null-space projection of the muscle activation (“Data”) and as the 95% percentile, over 500 repetitions, of the product of the null-space projection of the muscle activation, randomly shuffled during each repetition (“Random”). Values reported on the “Data” rows in bold were higher than the “Random” ones reported immediately below. The regression and the fitting were performed for data whose activation of each muscle was lower than the selected value.

Muscles Activation (%MVC)			10	20	30	40	50	60	70	80	90	100
Poses with a statistical correlation (%)	Major axis		8	61	85	91	95	95	97	98	98	98
	Minor axis		9	61	82	90	96	97	98	98	98	98
	Area		4	57	79	86	93	93	92	95	96	96
VAF (%)	Major axis	Data	**76**	**75**	**74**	**73**	**73**	**72**	**72**	**73**	**73**	**72**
		Random	69	55	48	44	41	37	35	34	33	33
	Minor axis	Data	49	41	40	41	42	44	45	**45**	**45**	**45**
		Random	67	58	54	51	49	48	45	44	44	43
	Area	Data	45	35	31	28	27	26	25	25	24	22
		Random	61	48	41	37	34	32	28	27	26	26

**Table 2:** VAF calculated with the axes and the volume of the stiffness ellipsoid approximated by the product of the null-space projection of the muscle activation, normalized to the ratio between the mean null-space projection of the muscle activation divided by the mean value of the ellipsoid axes or volume. In the table, both the VAFs calculated by fitting of the norm of the null-space projection obtained from data (“Data”) and the 95% percentile, over 500 repetitions, of the product of the null-space projection of the muscle activation, randomly shuffled during each repetition (“Random”) are reported. VAF reported in bold identifies “Data” values higher than “Random” values. VAF values reported with an asterisk (*) indicate a significant regression (*p* < 0.05) of the variable with the norm of the null-space projection of the muscle activation. The penultimate column reports the mean (std) values across participants. The last column reports the *p*-values of Student’s *t*-test between the VAF values reported in “Data” with respect to those reported in “Random”. Values reported in bold indicate a significant difference (*p* < 0.05).

Participant ID			1	2	3	4	5	6	7	8	Mean (Std)	*p*-Value
VAF (%) Baseline block	Axis 1 (Major)	Data	67 *	74 *	63 *	66 *	51 *	31 *	73 *	55 *	60 (14)	**0.019**
		Random	65	72	62	65	50	32	70	54	59 (13)	
	Axis 2 (Middle)	Data	66 *	74 *	62 *	65 *	47 *	32 *	72 *	55 *	59 (14)	0.826
		Random	67	72	63	66	49	31	70	54	59 (14)	
	Axis 3 (Minor)	Data	68 *	72 *	63 *	67 *	55 *	23 *	72 *	56 *	59 (16)	0.105
		Random	66	71	61	65	49	27	70	52	58 (14)	
	Volume	Data	66	72 *	62 *	65 *	49 *	22 *	71 *	54 *	58 (16)	0.741
		Random	66	71	61	64	48	26	72	51	57 (15)	
VAF (%) Perturbed blocks	Axis 1 (Major)	Data	85 *	87 *	85 *	**82**	77 *	81 *	86 *	**74**	82 (5)	**<0.001**
		Random	83	84	83	81	76	79	85	72	80 (4)	
	Axis 2 (Middle)	Data	86 *	86 *	85 *	83 *	75 *	82 *	86 *	74 *	82 (5)	**0.006**
		Random	83	84	83	81	76	80	85	72	80 (4)	
	Axis 3 (Minor)	Data	85 *	83 *	83 *	75 *	74 *	68 *	81 *	66 *	77 (7)	1.000
		Random	83	81	81	78	73	71	80	68	77 (5)	
	Volume	Data	86 *	84 *	84 *	76 *	73 *	69 *	**82**	66 *	77 (7)	0.885
		Random	83	82	82	78	74	71	80	69	77 (5)	

## Data Availability

The data presented in this study are available upon request from the corresponding author. The data are not publicly available due to privacy restrictions.

## Appendix A

The equation that approximates the force exerted by the active element (FA) given the length of the muscle ($l_{p}$) is as follows:

(A1) $$F_{A}=m\cdot F_{MAX}\cdot [-a\cdot {\left(\frac{l_{p}}{l_{ceopt}}\right)}^{2}+2a\cdot \frac{l_{p}}{l_{ceopt}}-a+1],$$

where $l_{ceopt}$ is the optimal length at which the muscle exerts the maximum force, $F_{MAX}$; and a=1width2, where width = 0.66 is the muscle width. The relation between the active isometric force and the muscle activation, m, was linearly modeled. The muscle activation was reported as a fraction of the Maximum Voluntary Contraction (MVC), which is the maximum contraction the muscle could exert. Hence, the muscle activation is between 0 and 1.

The relation between the force exerted by the parallel spring ($F_{p}$) and the length of the muscle belly is as follows:

(A2) $$F_{p}=k_{p}{\left[\max\left(0,l_{cerel}-\frac{l_{p0}}{l_{ceopt}}\right)\right]}^{2},$$

where $l_{cerel}=\frac{l_{p}}{l_{ceopt}}$ is the length of the muscle relative to its optimal length; $k_{p}$ is the stiffness of the non-linear spring in parallel with the active element, which was chosen such that, at $F_{p}=F_{MAX}$, $l_{cerel}=1+width$; and *l*_*p*0_ is the muscle belly relaxation length, which indicates the maximum length of the muscle for which the parallel spring does not exert any passive force. In this study, $l_{p0}$ was set equal to $l_{ceopt}$, as in [26].

The relation between the force exerted by the serial spring ($F_{s}$) and the length of the tendon is as follows:

(A3) $$F_{s}=k_{s}{\left[\max\left(0,l_{s}-l_{s0}\right)\right]}^{2}$$

where $k_{s}$ is the tendon stiffness, which was chosen such that, at $F_{MAX}$, $l_{s}=1.04\cdot l_{s0}$; $l_{s0}$ is the tendon slack length that indicates the maximum length of the tendon for which the serial spring does not exert any force; and $l_{ceopt}$, $l_{s0}$, and $F_{MAX}$ are muscle specific, and their values for the muscles used in this study were obtained from [26] and reported in Table 1.

The attachments on the bones are reported in Table 2. The BRD origin attach is on the humerus, and the insertion attach is on the radius. The TriLat origin attach is on the humerus, and the insertion attach is on the ulna. The PecMaj origin attach is in the sternal half of the clavicle, and the insertion attach is on the humerus. The DeltP origin attachment is in the scapula, and the insertion attachment is on the humerus. The biceps brachii short-head origin attachment is a coracoid process of the scapula, and the insertion attachment is on the radial tuberosity of the radius. The TriLat origin attach is in the scapula, and the insertion attach is on the ulna. The length of the humerus was set to be 0.30 m, while the forearm is set to be 0.25 m length. Both the humerus and forearm lengths were obtained from the Stanford VA upper-limb model developer for OpenSim^®^, which used the muscle characteristics reported in [26] and used in this study.

**Table A1:** Hill muscle parameters obtained from the literature and distance between the joint centers of rotation and the connection between the flexor (Flew) and extensor (Ext) muscles and the bones. The origin attach is intended as the distance between the connection of the origin side of the muscle with the bone, while the insertion attach is intended as the distance between the connection of the insertion side of the muscle with the bone.

	Elbow Mono-Articular Muscles		Shoulder Mono-Articular Muscles		Bi-Articular Muscles	
	Flex (BRD)	Ext (TriLat)	Flex (PecMaj)	Ext (DeltP)	Flex (BB)	Ext (TriLong)
$F_{MAX}$ (N)	261.33	624.30	364.41	259.88	435.56	798.52
$l_{ceopt}$ (m)	0.1380	0.1138	0.1442	0.1367	0.1157	0.1340
$l_{p0}$ (m)	0.1380	0.1138	0.1442	0.1367	0.1157	0.1340
$l_{s0}$ (m)	0.1726	0.0980	0.0028	0.0038	0.1923	0.1430
Scale factor	0.85	0.85	0.90	0.70	0.95	0.95
Scaled $l_{ceopt}$ (m)	0.1173	0.0967	0.1298	0. 0957	0.1099	0.1273
Scaled $l_{p0}$ (m)	0.1173	0. 0967	0.1298	0. 0957	0.1099	0.1273
Scaled $l_{s0}$ (m)	0.1467	0.0833	0.0025	0.0027	0.1827	0.1358
$k_{p}$ (N/m)	600	1433	600	1433	170	700
$k_{s}$ (N/m)	163 × 10^3^	390 × 10^3^	163 × 10^3^	390 × 10^3^	46 × 10^3^	191 × 10^3^
Origin attach (m)	0.130	0.022	0.042	0.110	0.037	0.010
Insertion attach (m)	0.200	0.190	0.071	0.056	0.039	0.041
$F_{MAX}$ (N)	261.33	624.30	364.41	259.88	435.56	798.52

The force-balancing equations for the mono-articular muscles acting on a joint are as follows:

(A4) $$F_{s}=F_{p}+F_{A},$$

(A5) $$\{\begin{array}{c} l_{mt}=l_{p}+l_{s}\\ l_{mt}=\sqrt{l_{1}{}^{2}+l_{2}{}^{2}-2\cdot l_{1}\cdot l_{2}\cdot \cos\beta }\;\;\;\;\;\;\;\;\;\;\;\;\;\;\\ m_{a}=\frac{2}{l_{mti}}\sqrt{\frac{P}{2}\left(\frac{P}{2}-l_{1}\right)\left(\frac{P}{2}-l_{2}\right)\left(\frac{P}{2}-l_{mti}\right)} \end{array},$$

where $l_{mt}$ is the length of the muscle; P is the perimeter of the triangle whose sides are $l_{1}$, $l_{2}$, and $l_{mt}$; $l_{1}$ is the origin attachment of the muscle to the bone; $l_{2}$ is the insertion attachment of the muscle on the bone; β is equal to the joint angle for flexor muscles and to its supplementary for extensor muscles; and $m_{a}$ is the moment arm.

The equations for the determination of the length, $l_{bt}$, and the moment arm of the bi-articular muscles with respect to the elbow ($m_{elbow}$) and shoulder ($m_{shoulder}$) joints are calculated as follows:

(A6) $$\{\begin{array}{c} l_{bt}=\sqrt{{\left(l_{2}\cdot \cos\left(\gamma \right)-l_{H}\cdot \cos\left(\beta_{1}\right)+l_{1}\right)}^{2}+{\left(l_{H}\cdot \sin\left(\beta_{1}\right)-l_{2}\cdot \sin\left(\gamma \right)\right)}^{2}}\\ \begin{array}{c} m_{elbow}=l_{2}\cdot \sin\alpha_{1}\\ m_{shoulder}=l_{1}\cdot \sin\delta \end{array} \end{array},$$

where $l_{H}$ is the length of the humerus; γ is equal to the sum of the elbow (α) and the shoulder (β) angles for the BB muscle and to the sum of their supplementary for the TriLat muscle; $\beta_{1}$ is equal to the shoulder angle for the BB muscle and to its supplementary for the TriLat muscle; and δ, $\alpha_{1}$, and c are as follows, respectively:

δ=asin(lH·sinβc)+asin(li2·sin(α−asin(li1·sinβ)c)c2+li22−2c·li2·cos(α−asinli1·sinβc)),

$$\alpha_{1}=\alpha +\beta +\delta -\pi ,$$

$$c=\sqrt{l_{i1}^{2}+l_{H}^{2}-2\cdot l_{i1}\cdot l_{H}\cdot \cos\beta }.$$
